# Optimal Path Planning Algorithm with Built-In Velocity Profiling for Collaborative Robot

**DOI:** 10.3390/s24165332

**Published:** 2024-08-17

**Authors:** Rafal Szczepanski, Krystian Erwinski, Mateusz Tejer, Dominika Daab

**Affiliations:** 1 Department of Automatics and Measurement Systems, Institute of Engineering and Technology, Faculty of Physics Astronomy and Informatics, Nicolaus Copernicus University, Wilenska 7, 87-100 Torun, Poland; erwin@umk.pl (K.E.); mattt@umk.pl (M.T.); 2 Department of Geomatics and Cartography, Faculty of Earth Sciences and Spatial Management, Nicolaus Copernicus University, Lwowska 1, 87-100 Torun, Poland; ddaab@umk.pl

**Keywords:** path planning problem, collaborative robot, velocity profile, nature-inspired optimization algorithm, pick-and-place, B-spline

## Abstract

This paper proposes a method for solving the path planning problem for a collaborative robot. The time-optimal, smooth, collision-free B-spline path is obtained by the application of a nature-inspired optimization algorithm. The proposed approach can be especially useful when moving items that are delicate or contain a liquid in an open container using a robotic arm. The goal of the optimization is to obtain the shortest execution time of the production cycle, taking into account the velocity, velocity and jerk limits, and the derivative continuity of the final trajectory. For this purpose, the velocity profiling algorithm for B-spline paths is proposed. The methodology has been applied to the production cycle optimization of the pick-and-place process using a collaborative robot. In comparison with point-to-point movement and the solution provided by the RRT* algorithm with the same velocity profiling to ensure the same motion limitations, the proposed path planning algorithm decreased the entire production cycle time by 11.28% and 57.5%, respectively. The obtained results have been examined in a simulation with the entire production cycle visualization. Moreover, the smoothness of the movement of the robotic arm has been validated experimentally using a robotic arm.

## 1. Introduction

In modern industries, optimizing the production cycle is one of the main ways to improve productivity. In the area of robotics, path planning optimization can enable operation at increasingly high velocities and, therefore, improve productivity [1,2,3]. In most cases, obtaining high velocities requires high accelerations, which can induce vibrations, resulting in a not-smooth trajectory [4]. The generation of a velocity profile for a predefined trajectory is another problem related to path planning. In this case, the trajectory is known in advance, and the problem is to achieve the shortest execution time with a particular focus on velocity, acceleration, jerk, and contour error limits. For this reason, connecting the path planning problem with the velocity profile generation is a non-trivial task.

From the application point of view, this problem is fundamental, and, recently, many researchers have taken into account the smoothness of path planning trajectory generation. Investigation of the path planning problem was performed for industrial robots [5], mobile robots [6], quadrotors [7], and autonomous underwater vehicles [8], and the most commonly used criteria were minimum-time [9], minimum-jerk [10], and minimum-energy [11]. In [12], the improved A* algorithm was proposed, and it has been successfully applied to plan the trajectory for the robot manipulator. An additional post-processing stage was used to optimize the resulting path by reducing the number of local paths and the path length. In [13,14], the path planning problem was solved using the ABC algorithm. The obtained results prove that the application of ABC can guarantee the shortest path. Meanwhile, the overall distance has only been minimized in the objective function, i.e., lacking the velocity, acceleration, and jerk limitation. The smooth movement of a point-to-point operation was proposed in [15,16]. In these approaches, the velocity profile is generated for start and end points to limit the velocity, acceleration, and jerk limitation. It allows for the execution of the movement in minimum-time. However, the trajectory has to be predefined. In [17], continuous-curvature path planning with obstacle avoidance was considered. To obtain a smooth trajectory, the S-shaped and half-S-shaped curves were investigated. The proposed continuous-curvature path planning algorithm allows for the receiving of a collision-free smooth trajectory. The minimum-time approach was not applied to the proposed algorithm.

The above-mentioned algorithms focus on only one of the aspects: minimum-time or the smoothness of the movement (related to minimum-jerk). For the manipulator, the collision-free trajectory has to be planned, and the limitation of motion variables has to be considered. Such an approach was proposed for a quadrotor motion planning system for fast flight in complex three-dimensional environments in [7]. The initial trajectory is based on minimum-time, and the additional task related to improving trajectory smoothness is applied. Due to the usage of two separate algorithms for trajectory planning and smoothing executing one after the other, there is no guarantee of obtaining the optimal trajectory with the required smoothness. For this reason, the optimization of path planning in consideration of both, i.e., minimum-time and minimum-jerk, has also been investigated in the literature [4,8,18]. In [4], optimal robotic trajectory planning subjected to kinematic and dynamic constraints was proposed. The application of the Multi-objective Ant Lion Optimizer was used to create two objective functions to minimize the total travel time and squared jerk torque rate. In such a case, the robot does not operate with the maximum jerk due to a compromise between the two connected objective functions during the optimization. A similar approach based on the B-spline curve was presented in [18]. The proposed method is based on a multi-objective optimization approach with two terms: minimum-time and minimum-jerk. The minimizing objective function was defined as the sum of the integral of the squared jerk and total execution time. In multi-objective optimization-based approaches, the velocity, acceleration, and jerk are not maximized to the limit values. Therefore, the total execution time is not minimal due to the balance between jerk limitation and the execution time.

In [8], a motion-planning algorithm based on the Particle Swarm Optimization algorithm that minimizes the traveling time of the slowest vehicle in the multiple-vehicle system was presented. The radial and tangential velocities and maximum linear velocity were considered as constraints within the optimization process. In such a case, the minimum-time was the main objective, and the velocity and acceleration were limited to their maximum values. However, there is a lack of jerk limitation. Moreover, the proposed method considers a simple environment without static or dynamic obstacles. Therefore, the problem of minimum-time optimization for two-dimensional path planning based on a four-point Bezier curve without jerk limitation seems non-universal.

The literature review of path planning algorithms shows that most methods take into account only one of two primary objectives: minimum-time or minimum-jerk. For the factory’s production rate, the most important path planning objective is to achieve the shortest production cycle time. On the other hand, guaranteeing path smoothness, which helps limit the velocity, acceleration, and jerk of the end effector, is indispensable, especially when liquids or delicate items are processed and moved. Therefore, the path planning algorithm has to reach the maximum allowable values of motion variables and still provide the shortest possible path length, taking into account the trajectory smoothness. In several papers, multi-objective path planning is considered [4,18]. However, the methodology, to the authors’ best knowledge, can be improved. The multi-objective optimization is based on connecting multiple assumptions into a single objective function. Such a definition for the optimization algorithm means that the obtained result will be a compromise between all objectives. For example, combining minimum-time and minimum-jerk objectives [4] provides a result where the jerk is not reaching its maximum allowable values during the execution of the trajectory, and then the time is not minimal. In order to achieve the shortest execution time and assumed smoothness of the trajectory, considering the velocity, acceleration, and jerk limits of the gripped item (in the particular case of a pick-and-place application), the limited variables should be maximized to their limitation values. For this reason, the path planning algorithm should consider the proper velocity profile of the evaluating trajectory. In this paper, the path planning algorithm for the pick-and-place application of a collaborative robot is considered. The proposed algorithm is divided into two parts, encapsulated into a single optimization process. These parts are a velocity profile generation to achieve the highest possible velocity, taking into account the motion limits, and a minimum-time path planning algorithm based on a nature-inspired optimization algorithm. The proposed algorithm uses a B-spline curve to obtain a smooth path to define the trajectory. The application of the nature-inspired optimization algorithm to solve the path planning problem is supported by the superior properties of these types of optimization algorithms, and they have an essential and influential role in addressing the path search problem [19,20]. In this paper, the application of the Artificial Bee Colony (ABC) optimization algorithm is proposed. The ABC algorithm is inspired by the foraging behavior of honey bees, and it has been one of the most frequently applied nature-inspired optimization algorithms over the last few years [21]. The algorithm is divided into three phases: the employed bee phase, the onlooker bee phase, and the scouts phase. The first and the second are responsible for the exploitation and the exploration of the search space, while the third one allows for the avoidance of the stagnation in the local minimum. The Artificial Bee Colony algorithm has been selected due to its superior performance in solving engineering problems [22] and the experience of the authors with the implementation of various optimization problems. It should be noted that the proposed nature-inspired optimization algorithm can be replaced with the one preferred by the applicator. The related literature shows that the results obtained do not allow for the simultaneous consideration of both jerk and path smoothness. The solution proposed in this paper shows the possibility of achieving these goals simultaneously by utilizing a nature-inspired path optimization algorithm and a velocity profiling algorithm applied during the optimization process.

The authors’ contribution in this paper is summarized in the following points.

- The authors presented and experimentally verified a solution for the pick-and-place problem for a collaborative robot with a particular emphasis on providing smooth motion.
- A velocity, acceleration, and jerk profile generation algorithm with a particular emphasis on limiting the allowable chord error for a B-spline trajectory was proposed.
- A nature-inspired optimization algorithm was utilized to select the optimal B-spline path that ensures minimum-time execution and takes into account motion limitations and the smoothness of the trajectory.
- Experimental verification was performed and presented to show the smoothness of the robot operation using the proposed algorithm.

## 2. B-Spline Path Interpolation

Robots are usually programmed by defining a set of points through which the end effector should travel while performing the programmed task. In the simplest case, the path of the end effector between these points is piecewise linear. This leads to discontinuous derivatives of the path with respect to the path parameter (i.e., distance). Discontinuity of path derivatives would result in significant sudden changes in velocity if the path would be traveled with constant velocity. In such cases, either the path is smoothed by the robot controller, or the end effector comes to a complete stop at each destination point. To remedy this, the end effector path can be defined as a polynomial spline with guaranteed continuity of certain derivatives. Such a path results in smoother velocity as well as higher position time derivatives like acceleration and jerk, and less strain on the mechanical elements and drives of the robot.

In order to guarantee that derivatives up to a certain level are continuous, a B-spline curve can be used as the robot’s end-effector path definition. B-splines are polynomial spline curves defined by the curve order, a knot vector, and a series of control points. Control points define the shape of the curve in Cartesian space. The knot vector determines curve breakpoints between polynomial segments. The curve parameter *u* is a unitless, normalized parameter of the curve which describes the desired end-effector path. The parameter unambiguously defines a position on the curve. Value of 0 indicates the start of the path and a value of 1 indicates the end of the path. When interpolating the path to compute desired coordinates of the end effector at each time step, a particular value of u has to be computed. The parameter is usually not equivalent to the arc length of the curve, but the increment of the parameter at each time step has to correspond to an increment of the arc length which is proportional to the desired end-effector velocity at this particular time step. Cartesian coordinates of a point on the curve, which correspond to a given value of u, are determined by curve evaluation using de Boor’s algorithm [23]. This algorithm can also provide the curve’s derivatives. One of the main advantages of B-spline curves is their definition by control points, which influence the curve shape only locally, and changing their location has an easily predictable influence on the curve’s shape. Furthermore, a curve of a given degree *d* has a guaranteed continuity of $C^{d-1}$ at the breakpoints. This means that, for a cubic (3rd degree) curve, both the first and second derivatives with respect to the curve parameter are continuous. Due to these traits, this curve type is often used to define paths in motion control applications such as CNC machines, robotics, and mobile robotics [24,25].

In motion control applications, the B-spline is evaluated successively from start to end, reflecting the travel of the end-effector along the curve. Each point is evaluated in constant time intervals. Cartesian coordinates of successive points are transformed using inverse kinematics to joint positions and then sent to the joint servo drives. The distance between each point is, therefore, proportional to the end-effector velocity tangent to the toolpath. To control the effector velocity, a velocity profile has to be determined, and the curve has to be interpolated so that the distance between each point corresponds to the predetermined velocity profile.

The relationship between the increment of the curve parameter and the distance increment is non-linear. This means that an identical increase in parameter value corresponds to a different incremental distance depending on the current location on the curve. This relationship is also unique for every curve. At each interpolation step, an approximation has to be performed that determines the required value of parameter increment. A second-order Taylor expansion method is used [26], with the next curve parameter determined by the following formula:

(1) $$u_{i+1}=u_{i}+\frac{1}{\left|\right|C^{\prime }\left(u_{i}\right)\left|\right|}\Delta s+\frac{1}{2}\frac{-C^{\prime }\left(u_{i}\right)\cdot C^{\prime \prime }\left(u_{i}\right)}{\left|\right|C^{\prime }\left(u_{i}\right){\left|\right|}^{4}}{\Delta s}^{2},$$

where $u_{i+1}$ is the next parameter value, $u_{i}$ is the current parameter value, Δs is the desired distance increment equal to $V_{i}\cdot \Delta T$, and $C^{\prime }\left(u_{i}\right),C^{\prime \prime }\left(u_{i}\right)$ are the first and second derivative of the B-spline path with respect to the curve parameter. The quantity $V_{i}$ is the current value of velocity, which is determined by evaluating the velocity profile.

## 3. The Proposed Path Planning Algorithm

The definition of the path planning problem is to ?find a collision-free motion between an initial (start) and a final configuration (goal) within a specified environment? [27]. In this paper, an additional term is taken into account in the path planning problem: the path smoothness with limited velocity, acceleration, and jerk profiles. In many applications, the maximum values of these variables have to be limited, e.g., moving a delicate item, a container with a liquid, or collaboration with a human coworker. Regardless of the limitations and assumptions, the most important indicator in a factory is the production rate. For this reason, the execution time of the production cycle has to be minimized. The most commonly utilized approach of the trajectory planning algorithms focuses on the shortest path [13,14,28]. However, generating the shortest possible path does not always result in obtaining the shortest possible execution time. If the shortest path is optimized as point-to-point moves, then the robot has to stop at each control point to limit the acceleration and jerk. An attempt to go through breakpoints of a piecewise linear path would result in abrupt changes in velocity, acceleration, and jerk due to discontinuities of point-to-point path derivatives. In such a case, a longer but smoother path defined as a polynomial B-spline can be executed without stopping and, therefore, provide a shorter execution time due to the higher velocity at the arc.

The proposed path planning algorithm contains two methods: the Artificial Bee Colony optimization algorithm to ensure the global optimality of the obtained path, and a velocity profiling algorithm to determine the velocity, acceleration, and jerk profiles for the input path defined as a B-spline curve. Such a connection allows for the obtaining of optimal, smooth, collision-free, B-spline paths for the pick-and-place applications.

### 3.1. Velocity Profiling Algorithm

In order to increase the tracking accuracy of the end-effector path, a proper velocity profile is required for interpolation. The velocity profile defines the value of end-effector velocity as a function of time or some other parameter, such as the path position. Usually, acceleration and jerk are limited so that the robot’s joints work within their capabilities. This allows for keeping the path tracking accuracy within an acceptable range and alleviates excessive vibration. On the other hand, the path velocity should be as high as possible to shorten work cycles and increase productivity. Direct limitation of joint constraints by profiling the path velocity is a difficult optimization problem due to the complex kinematics of an articulated robotic arm. An alternative approach is to limit only the velocity, acceleration, and jerk of the end-effector velocity tangent to the toolpath using a jerk-limited velocity profile called an S-curve [16]. Large changes in velocity, which affect path-tracking accuracy, usually occur in fragments of the toolpath with increased curvature. Therefore, selectively decreasing the velocity in such critical fragments of the desired end-effector path is beneficial. Other parts of the path are executed with the maximum velocity to shorten the execution time.

The velocity profile for the robot’s end effector is planned between curvature-critical points as a sequence of S-curve profiles. At these points, the B-spline path reaches high curvature values (sharp corners), which require the velocity to be reduced to satisfy the velocity, acceleration, and jerk constraints. In order to determine these points, the B-spline curve derivatives are first evaluated at constant intervals of the curve parameter *u* and then the velocity limit function $V_{lim}$ is evaluated using the following equations [24]:

(2) $$\kappa =\frac{1}{\rho }=\frac{\left|\right|C^{\prime }\left(u\right)\times C^{\prime \prime }\left(u\right)\left|\right|}{\left|\right|C^{\prime }\left(u\right){\left|\right|}^{3}},$$

(3) $$V_{lim}=\min\left(V_{max},\frac{2\sqrt{2\rho \epsilon -\epsilon^{2}}}{\Delta t},\sqrt{\frac{A_{max}}{\kappa }},\sqrt[{3}]{\frac{J_{max}}{\kappa^{2}}}\right),$$

where κ is the curvature, ρ is the curvature radius, ϵ is the maximum allowable chord error, Δt is the interpolation period, $A_{max}$ is the maximum acceleration, and $J_{max}$ is the maximum jerk.

The chord error velocity limit stems from approximating the curve locally with an arc with a radius equal to the curvature radius at that point. The perpendicular distance between the arc and a line between consecutive interpolation points is treated as the chord error. The chord error is a parameter that determines how closely the interpolated end-effector trajectory follows the desired path defined as a B-spline curve. This, in turn, influences how much the velocity is reduced for a given curvature of the end-effector path. Acceleration and jerk-related velocity limits are derived from the maximum centripetal acceleration and jerk for a given curvature. If a velocity limit value for a certain value of *u* is lower than two neighboring values, this point is flagged as a coarse curvature-critical point. These coarse minima are then refined using Brent’s method [29,30] to find the *u* parameter values for which the velocity limit reaches a local minimum. Obtaining the precise location of curvature-critical points is required to properly plan the velocity between them.

First, the full seven-phase velocity profile is planned between the critical points for a given start velocity $V_{start}$, end velocity $V_{end}$, maximum velocity $V_{max}$, acceleration, and jerk. If the segment length is long enough to incorporate the full seven-phase S-curve velocity profile, the duration of the constant velocity phase is adjusted so that the total displacement derived from the profile is equal to the distance between the current critical points. The segment length may be too short to achieve the maximum velocity with the given acceleration and jerk constraints. This is determined by evaluating the total distance $S_{tot}$ covered by the acceleration and deceleration profiles. If this distance is less than the segment length $S_{seg}$, the constant maximum velocity phase is added. If the distance covered by full acceleration and deceleration profiles is greater than the segment length, the constant velocity phase is then omitted and the maximum velocity is unreachable. The peak velocity value $V_{peak}$, which is lower than the maximum velocity, has to be determined numerically. This value is computed iteratively so that the total actual displacement (length of travel) of the end-effector in the current segment ($S_{tot}$) resulting from the velocity profile is equal to the desired displacement, which is equal to the arc-length of the path between the path’s curvature-critical points ($S_{seg}$). A bisection search [30] is performed in order to find the $V_{peak}$ for which the total acceleration and deceleration profile distance is less than or equal to the segment length. At each iteration, the search span is halved until the change in velocity in each iteration is less than a specified value (0.01 mm/s was used), and the total segment length is less than or equal to the segment span. The peak velocity is computed at each iteration using the following equation:

(4) $$V_{peak_{k}}=\left\{\begin{array}{c} V_{{peak}_{k-1}}+\frac{\Delta V_{k}}{2^{k}}\;S_{tot}\leq S_{seg}\\ V_{{peak}_{k-1}}-\frac{\Delta V_{k}}{2^{k}}\;S_{tot}>S_{seg} \end{array}\right.,$$

where $\Delta V_{k}$ is the current velocity search span between the peak velocity from the previous iteration and start or end velocity limit (whichever is larger), and *k* is the iteration counter starting from 1. The initial value of $V_{peak}$ is equal to $V_{max}$. The current search span is computed using the following equation:

(5) $$\Delta V_{k}=V_{{peak}_{k-1}}-\max\left(V_{start},V_{end}\right).$$

**Figure 1:**
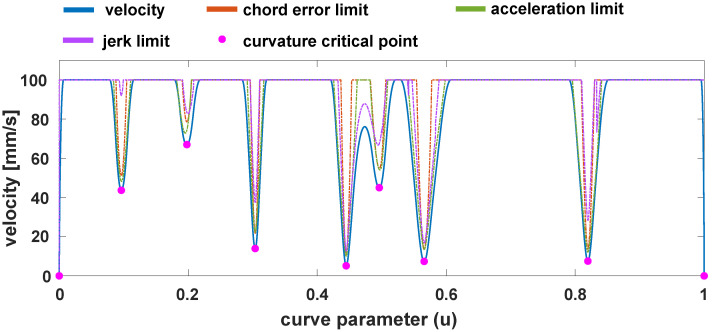
Example velocity profile planned between curvature-critical points.

In each iteration of *k*, the velocity profile is recomputed for the new value of $V_{peak}$. The peak velocity is adjusted until the distance of the profile is less than the total length of the segment. The velocity profile generation algorithm is repeated for each segment of the B-spline end effector path until all segments have their respective velocity profiles generated. The velocity planning algorithm is described in more detail in the authors’ previous paper [31]. An example velocity profile with limits imposed in high-curvature fragments is presented in Figure 1. The dashed lines in the figure indicate limits imposed by the components of the velocity limit function $V_{lim}$ presented in Equation (3).

The generated velocity profile is applied to the B-spline path, which is optimized using a nature-inspired optimization algorithm.

### 3.2. Path Optimization Using Nature-Inspired Optimization Algorithm

The path optimization problem is usually defined as to minimize the path execution time. This can be defined by a function $T(\vec{x})$ which returns the trajectory execution time for an input control point vector $\vec{x}$. Each control point in the vector is comprised of X, Y, Z coordinates which together define a B-spline path of the end effector. The optimization problem is usually subject to constraints. These are the variable bounds, which limit the coordinates of the optimization variables (control points). This is defined as

(6) $$ub_{i}\geq x_{i}\geq lb_{i},\;\;\;\;\;\;\forall i=1\ldots \left(3\cdot N\right),$$

where *N* is a number of B-spline control points, while (3·N) is the problem dimensionality due to the path being planned in three dimensions, and $\vec{ub}÷\vec{lb}$ are the upper and lower bounds for the solution search space. In order for the robot to avoid obstacles and restricted spaces, further constraint violation functions are introduced:

(7) $$v_{R}\left(\vec{x}\right)=0,\;\;\;\;v_{W}\left(\vec{x}\right)=0,\;\;\;\;\;\;v_{Oj}\left(\vec{x}\right)=0,\;\;\;\;\;\;\forall j=1\ldots M,$$

The violation functions $v_{R}\left(\vec{x}\right)$, $v_{W}\left(\vec{x}\right)$, and $v_{Oj}\left(\vec{x}\right)$ indicate that the minimum allowable distance to the robot, worker area, and obstacle, respectively, are violated. *M* is the number of obstacles taken into account during the path planning process. The violation functions are generally defined as

(8) $$v\left(\vec{x}\right)=\left\{\begin{array}{cc} d_{\mathrm{violation}} & \mathrm{if}\;\mathrm{safety}\;\mathrm{area}\;\mathrm{is}\;\mathrm{violated}\\ 0 & \mathrm{otherwise} \end{array}\right.,$$

where $d_{\mathrm{violation}}$ is the value of how much the trajectory defined by $\vec{x}$ violates the analyzed object or safety area (in the case of the worker).

The T is related to the generation of velocity, acceleration, and jerk profiles for the B-spline trajectory. The application of the velocity profiling algorithm (Section 3.1) provides a velocity profile, which can be used to interpolate consecutive position set points for the robot until the final point is reached [32]. After such an interpolation, the overall time required to execute the trajectory with velocity, acceleration, and jerk limitations is obtained. At this point, the solution $\vec{x}$ is profiled during objective function evaluation, and the motion variables are not considered as constraints in the optimization process.

To solve the optimization problem, which is to achieve the shortest possible execution time and collision-free movement, a nature-inspired optimization is used. The Artificial Bee Colony (ABC) algorithm has been selected due to the authors’ experience with implementing various engineering optimization problems and solving them using the mentioned optimization algorithm. However, the proposed nature-inspired optimization algorithm can be replaced with the one preferred by the applicator. It is divided into three phases: employed, onlooker, and scout bees. These phases allow for good exploitation and exploration and avoid the stagnation in the local minimum. The important gain of the usage of this algorithm is that its formulas can take into account the constraint function in the selection of new solutions to evaluate. It should be noted that other popular swarm-based optimization algorithms, e.g., the Particle Swarm Optimization algorithm, Genetic algorithm, Ant Colony Optimization algorithm, and Grey Wolf Optimizer, are not equipped with this particular solution. In order to use a nature-inspired optimization algorithm to solve a constrained optimization problem, a constraint handling method has to be applied. In the proposed approach, Deb’s rules are used. The description with a deep analysis of ABC combined with Deb’s rules can be seen in [33]. Due to the ABC algorithm maximization of the objective function, the objective function is calculated by the following formula:

(9) $$F\left(\vec{x}\right)=\frac{1}{1+T(\vec{x})},$$

while the overall violation function proposed by the authors is defined as follows:

(10) $$V\left(\vec{x}\right)=\frac{1}{1+\mathrm{MIN}\left(v_{R}\left(\vec{x}\right),v_{W}\left(\vec{x}\right),\left\{v_{Oj}\left(\vec{x}\right),\;\;\;\;\forall j=1\ldots M\right\}\right)}.$$

The algorithm of solution evaluation is presented in Algorithm 1.
**Algorithm 1** Objective function evaluation for ABC  **Input:** $\vec{x}$  **Output:** F, V
  1:  Get B-spline control points from the input $\vec{x}$  2:  Get T by generation velocity, acceleration and jerk profiles  3:  Calculate F using Equation (9)  4:  Divide the B-spline into check points (bscp)  5:  Set V to 0  6:  **for all** p in *bscp* **do**  7:      Set $d_{R}$ to distance from *p* to robot’s mounting position  8:      Set $d_{W}$ to distance from *p* to coworker’s position  9:      **for** j=1 to *M* **do** 10:          Set $d_{O_{j}}$ to distance from *p* to $O_{j}$ 11:      **end for** 12:      Clear all distances so that *p* does not violate violation area 13:      Set V to MIN(V, $d_{R}$, $d_{W}$, $\left\{d_{O_{j}},\;\;\;\;\forall j=1\ldots M\right\}$) 14:  **end for**

**Figure 2:**
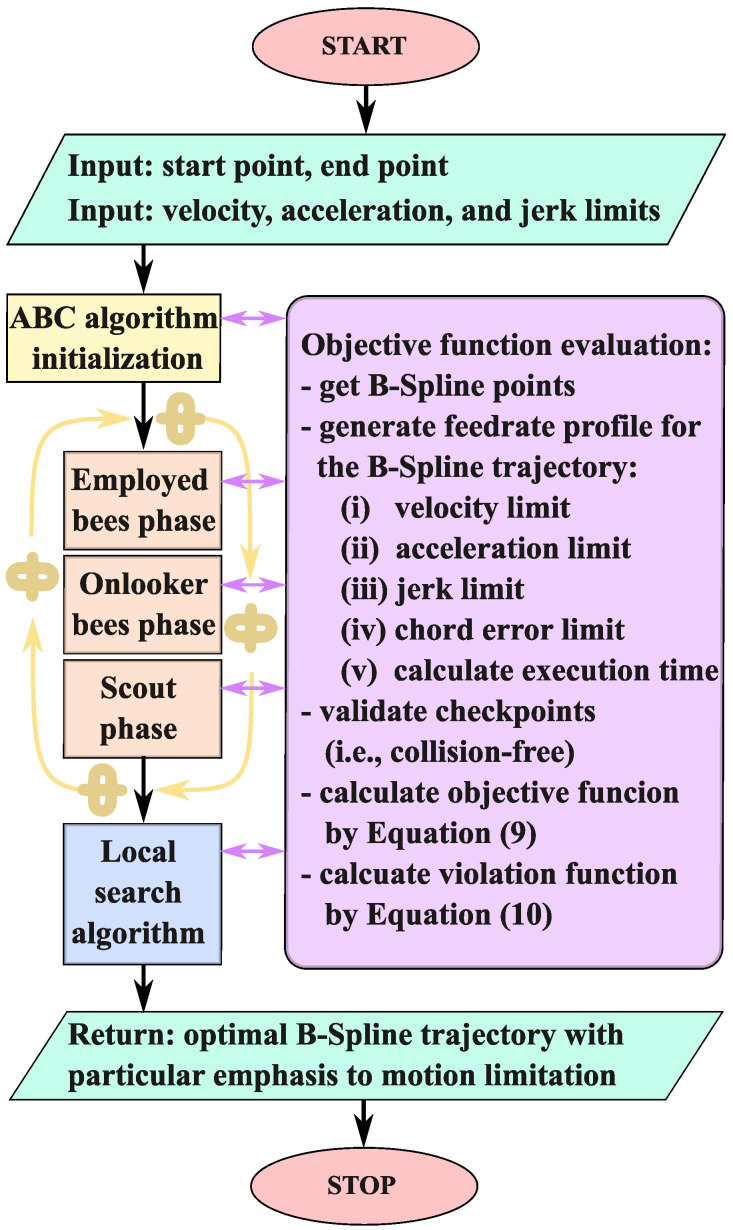
The general block diagram of the proposed trajectory optimization procedure.

It should be noted that the collision-free verification of path (P) is determined using the predefined number of checkpoints $bscp=[p_{1},p_{2},\ldots ,p_{\left|bscp\right|}]\in P$, equal to |bscp|=50 in this particular case. The path is defined as collision-free (V=0) if none of the points $p_{i}\in \left[1,2,\ldots ,|bscp|\right]$ violate the object safety area. The nature-inspired optimization algorithms are global optimization algorithms. Due to this, these algorithms, including the Artificial Bee Colony algorithm, have to provide good exploration to find the area of the global minimum and good exploitation to provide a solution close to or exactly equal to the global minimum point. Such a complete exploration and exploitation requires relatively high computational effort. One solution is related to improving exploitation based on specific operations for agents closest to the best found solution [34,35,36]. Another approach is the connection of a global optimizer (i.e., nature-inspired optimization algorithm) and a local one [37,38,39]. In the case of the local optimization algorithms, exploiting a selected area around the initial guess allows for a significantly improved solution with low computational costs. In this paper, the hybrid optimization based on the Artificial Bee Colony algorithm and Nelder–Mead simplex (direct search) method [30] was used to improve the exploration–exploitation balance in the optimization process. The general block diagram of the proposed trajectory optimization with a particular emphasis on the motion limitation is presented in Figure 2.

## 4. Example Application

In this section, the application of the proposed path planning algorithm is evaluated in a real-world problem, which is the optimization of the production cycle with the pick-and-place collaborative robot.

**Figure 3:**
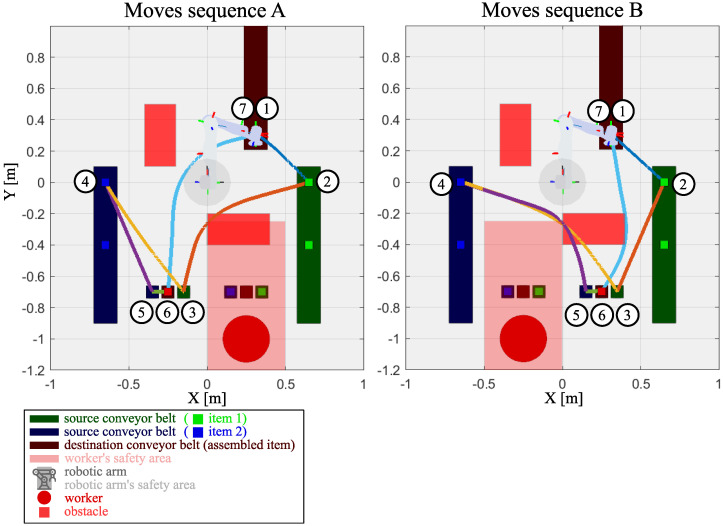
Optimal production cycles obtained by the proposed algorithm. Moves sequence A: coworker right position, Moves sequence B: coworker left position.

The collaborative robot is used as a pick-and-place machine to move items that are delicate and/or contain a liquid. The robot is responsible for providing a human coworker with two parts to assemble and then moving the assembled part to the outgoing conveyor belt. Due to this, the restrictions for the robot movement are as follows: (i) velocity, acceleration and jerk have to be limited to 100 mm/s, 100 mm/s^2^, and 150 mm/s^3^, respectively, (ii) the safety area of the coworker cannot be violated, (iii) smoothness in terms of a low trajectory curvature is necessary (i.e., $\epsilon =25\cdot 10^{-7}$; see the description in Section 3.1), (iv) a collision-free path is obligatory. In order to increase the production rate, the coworker works in two workspaces alternately. The robot puts the next parts to assemble and takes away the previously assembled item, while the coworker works in the opposite workspace. The entire production cycle, divided into two move sequences related to coworker position, can be seen in Figure 3, where the next pick-and-place positions are numbered in sequence. The problem is to obtain the shortest production cycle, taking into account constraints, and the proposed trajectory optimization method is applied to solve it.

### 4.1. Optimization Setup

Each trajectory is created by B-spline with the following settings: 8 control points, cubic shape (B-spline order set to 4). Two control points are always predefined as the start and end points. Due to this, the optimization procedure considered the manipulation of 6 control points with X, Y, and Z coordinates (the problem dimensionality was equal to (3·6)). The proposed trajectory optimization algorithm was executed 12 times in order to obtain a smooth, collision-free, and minimum-time B-spline trajectory. The used ABC parameters are listed in Table 1.

### 4.2. Comparison and Quality Indicators

The most common approach for the path planning of the robotic arm is point-to-point movement, where the points are user-defined. In such a case, the trajectory length has the highest importance, and the length is as short as possible. However, the analyzed problem of velocity, acceleration, and jerk limitation for point-to-point movement provides the requirement of stopping at each point. The reason is related to the discontinuity of such a trajectory. Nevertheless, this approach is commonly used in the industry, where the precision of movement is required (i.e., G01 from G-codes standard). Due to this, such an approach will be used as a comparison to the proposed approach. To satisfy the motion limitations, the jerk-limited velocity profile (called an S-curve) is utilized for a fair comparison.

Moreover, the state-of-the-art algorithm, which is a rapidly explored random tree (RRT) in the RRT* variant [40], has been used for comparison with the proposed approach. The provided path has been approximated by the B-Spline curve. Next, the same jerk-limited velocity profile has been applied for a fair comparison.

To provide more quantitative indicators than the execution time of the trajectory, the path quality indicators proposed in [41] were calculated to provide a quantitative comparison. The first one is the most common indicator, which is the path length. It can be expressed as

(11) $$\mathrm{length}\left(P\right)=\sum_{i=1}^{|P|-1}d\left(p_{i},p_{i+1}\right),$$

where $P=(p_{1},p_{2},\ldots ,p_{n})$ is the vector of the trajectory points, |P| determines the number of poses ($p_{i}\in \left[1,2,\ldots ,n\right]$), and $d(p_{i},p_{i+1})$ is a function providing the distance between two consecutive reference position points.

The smoothness of the trajectory can be expressed as the mean value of changes in the movement direction, determined as the orientation angle ($\theta_{i}$), which is the angle between two consecutive trajectory points [41]:

(12) $$S\left(P\right)={\left(\frac{1}{|P|-2}\sum_{i=1}^{|P|-1}{\left(\theta_{i}-\theta_{i+1}\right)}^{2}\right)}^{\frac{1}{2}},$$

### 4.3. Results

Each experimental test sequence consists of six moves. The sequence starts when item 1 is present on the green conveyor belt pick-up point (2). The arm picks up the item and places it in the manual work area (3). Then, the arm moves to pick up item 2 from the blue conveyor pick-up point (4) and places it in the manual work area (5). The worker then carries out the manual combination of the two items and places the resulting item in the pick-up point (6). After the worker moves away to the second manual work area, the robot picks up the ready item and places it on the red conveyor (6) and starts the next sub cycle when the next item 1 is ready for pick up. When the manual work is carried out, the robot picks up and places items at the free manual worker’s area. Hence, the two move variants A and B depend on the current position of the manual worker. The paths are generated so that any obstacles and worker-safe areas are not violated. Each robot arm move for two variants has been optimized to achieve the minimum time and obstacle avoidance. Each optimization took around 2 h, while the entire production cycle optimization took 24.75 h. It should be noted that the optimization process is performed only once, and the cooperative robot can move without time-consuming trajectory planning tasks. The obtained optimal trajectories for the collaborative robotic arm are presented in Figure 3. The results are divided into two move sequences related to the coworker’s position. These sequences provide the entire production cycle by executing one after the other.

Every obtained trajectory successfully avoided the obstacles and coworker violation area. One can see that the obtained trajectories are smooth to reduce their curvature and maximize the movement. All the velocity, acceleration, and jerk constraints have been met successfully. The executing times and path quality indicators of individual trajectories have been summarized in Table 2. Each trajectory is marked as a moves sequence from Figure 3 (A or B) and start–end points, i.e., A 1–2, A 2–3, etc. It can be seen that the smoothness indicator is consistently small for all optimal moves and those provided by the RRT* algorithm. This is caused by both methods using a B-Spline curve with jerk-limited profiling. For manual moves, the indicator is either zero (moves sequence over a straight line) or has a much higher value when the direction of movement changes. These sudden changes in direction cause a sudden change in velocity, which has a detrimental effect on motion accuracy and necessitates a large decrease in velocity. The obtained execution time of the entire production cycle realized using optimal trajectories is equal to 117.2 s, while this time using the point-to-point and RRT* algorithms with jerk-limited trajectories are equal to 132.1 s and 276.0 s, respectively. The proposed method realizes the same velocity, acceleration, and jerk limitation and decreases the entire production cycle time by 11.28% in comparison with point-to-point operation and 57.5% in comparison with RRT*.

**Figure 4:**
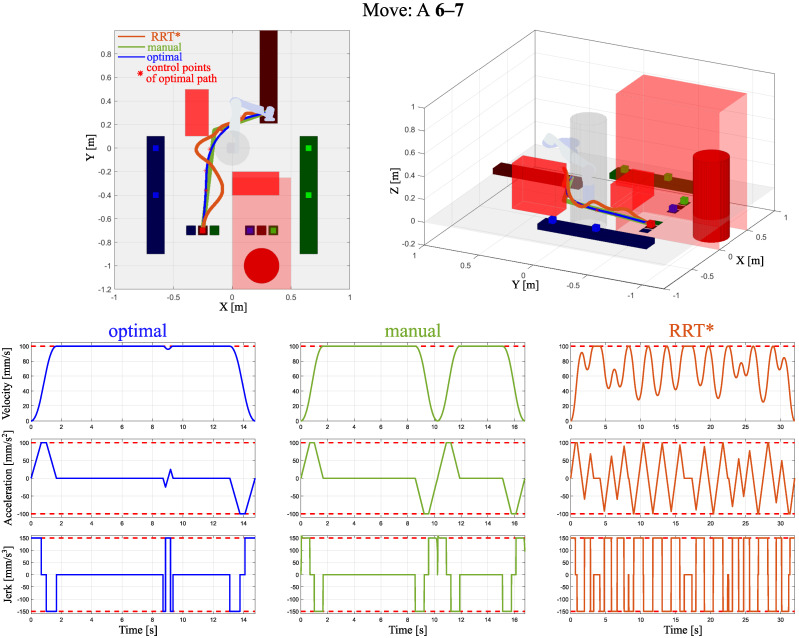
Obtained trajectories by the proposed path planning algorithm, named optimal, point-to-point method, and the RRT* algorithm, extended by their velocity, acceleration, and jerk profiles for move A 6-7.

**Figure 5:**
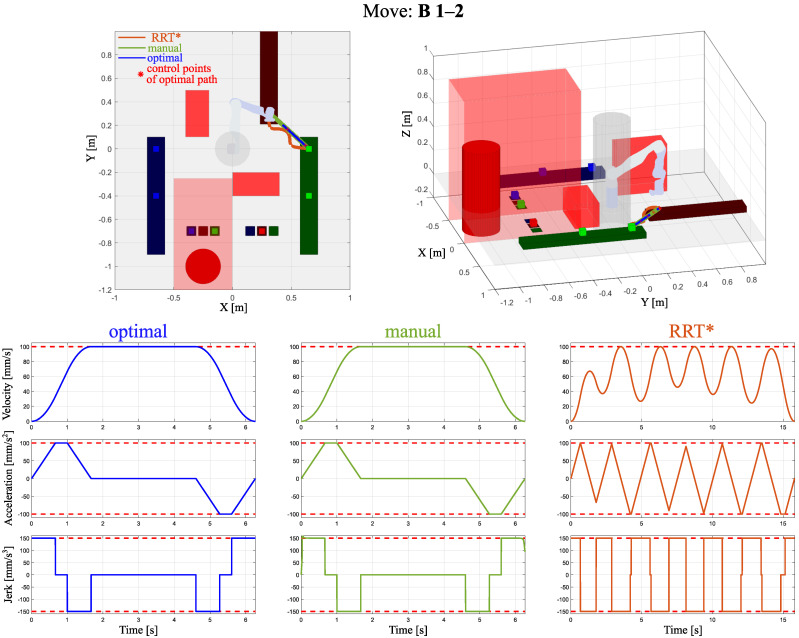
Obtained trajectories by the proposed path planning algorithm, named optimal, point-to-point method, and the RRT* algorithm, extended by their velocity, acceleration, and jerk profiles for move B 1-2.

**Figure 6:**
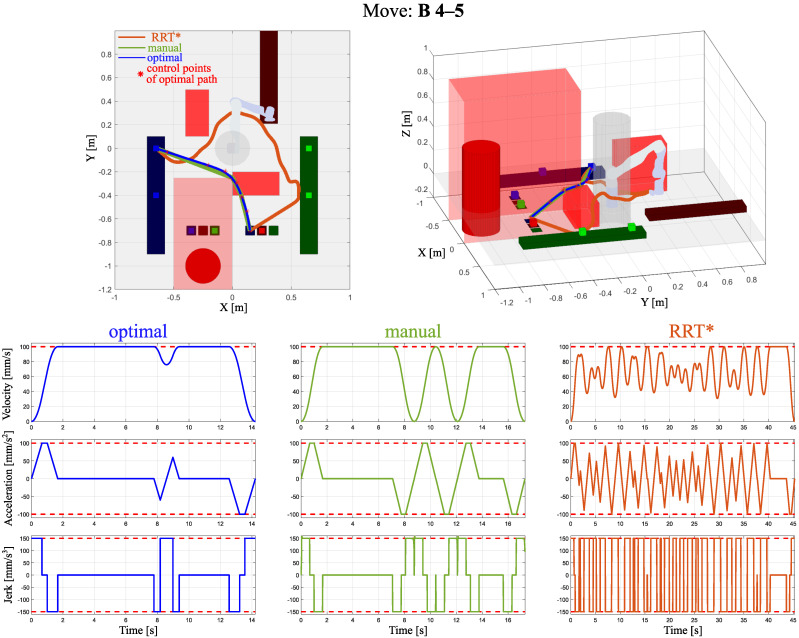
Obtained trajectories by the proposed path planning algorithm, named optimal, point-to-point method, and the RRT* algorithm, extended by their velocity, acceleration, and jerk profiles for move B 4–5.

In Figure 4, Figure 5 and Figure 6, three examples of optimized trajectories compared with point-to-point movements and the RRT* algorithm with velocity, acceleration, and jerk profiles are presented. One can see that the proposed path planning algorithm with built-in velocity profiling provides the solution without extreme decelerations. The RRT* algorithm focuses on finding the feasible path from the start to goal positions. It does not include the smoothness of the path. In such a case, maintaining the considered acceleration and jerk limits results in a highly oscillating velocity. Compared with the point-to-point operation, the proposed optimal trajectory based on B-spline minimizes extreme decelerations by insignificantly increasing the path length. This notable improvement has been reached by connected path planning as an optimization problem with a novel computationally efficient method of velocity profiling.

**Figure 7:**
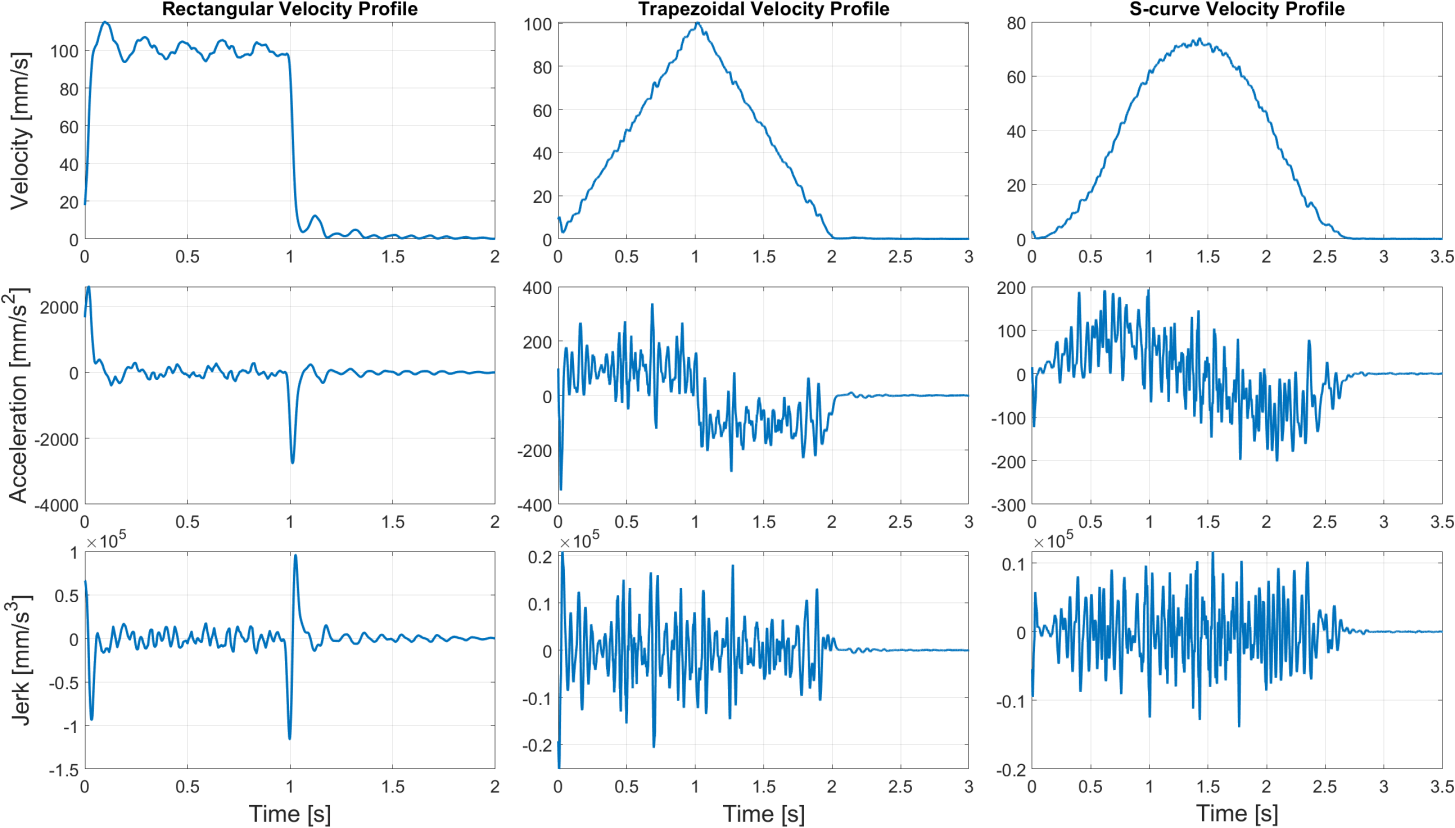
Experimental verification of different velocity profiles for 1 kg load.

The additional experimental verification for different velocity profiling methods has been provided, and it is presented as a video within Supplementary Files (Video S1: Robotic Arm profiling). The quality indicators of the movement have been summarized in Table 3. The experiments have been conducted using a Kinova Gen3 robotic arm with water in an open container and solid items with different weights. In the case of liquid, the rectangular, trapezoidal, and S-curve velocity profiles have been compared. Rectangular profiling exhibits the least smoothness of the robotic arm movement. Trapezoidal profiling reduced the value of the acceleration, providing a solution with relatively smooth movement. However, the vibrations related to unlimited jerk cause waves to form on the liquid surface during the beginning and ending phases of the movement. S-curve profiling, which is proposed in this paper, provides the smoothest movement with limited acceleration and jerk values. This significantly decreases disturbance on the surface of the liquid and minimizes the risk of spilling. Similar results can be observed in Table 3 for solid items. For the 1 kg solid item movement, the velocity, acceleration, and jerk have been presented in Figure 7. It should be noted that a real experiment provides a highly noisy measurement of velocity, and the acceleration/jerk has been calculated numerically. In such a case, the values exceed the assumed ones. However, the proposed method provides the smoothness operation. One can see that the proposed algorithm provides superior results in the form of motion smoothness regardless of the transported items.

## 5. Conclusions

A minimum-time path planning algorithm with a particular focus on velocity, acceleration, and jerk limits was proposed in this paper. The Artificial Bee Colony nature-inspired optimization algorithm was applied to ensure the minimum-time execution of the B-spline trajectory, while the proposed velocity profiling algorithm limits the chord error, velocity, acceleration, and jerk. The proposed path planning algorithm is based on a single-objective optimization procedure along with a novel constraint violation formula. The proposed solution does not require the definition of a multi-optimization problem or execution of a post-optimization procedure, which ensures that the found solution is a minimum of the defined problem. Therefore, the minimization of the production cycle time is achieved without requiring a compromise between different objectives, such as the minimization of time and minimization of jerk. In the proposed approach, the only indication for optimization is to minimize the production time, and the additional constraints are included separately. The application of the proposed approach has been evaluated by simulating a real-world problem, which is a pick-and-place operation of a collaborative robot in a dynamic environment. The obtained production cycle allows for keeping the smoothness of the trajectories and the minimum-time of the entire cycle, with particular care for velocity, acceleration, and jerk limits for the safe movement of delicate items. The proposed path planning algorithm has been compared with point-to-point operation and the RRT* algorithm. The proposed path planning algorithm significantly outperforms the state-of-the-art solutions in terms of the path length and production cycle time. Moreover, the obtained trajectory with the proposed velocity profiling (S-Curve) has been compared with trajectories with rectangular and trapezoidal profiling for four different transported items (including the liquid) by the Kinova Gen3 robotic arm to present its robustness in terms of smoothness. A Supplementary File (Video S2: Simulation) were added, which illustrates the capability for smoothing the trajectory to reliably transport liquids without spilling.

## Figures and Tables

**Table 1:** Artificial Bee Colony parameters.

Parameter	Value
No. of optimized parameters (*D*)	3·6
No. of colony size (NP)	32
No. of food sources (FN)	NP/2
Control parameter (limit)	FN·D
Scout production period (SPP)	FN·D
Modification rate control parameter (MR)	0.8
Number of iterations (*N*)	$10^{4}$
Lower-bound ÷ Upper-bound (lb÷ub) X and Y axes	−1÷1
Lower-bound ÷ Upper-bound (lb÷ub) Z axis	0÷1

**Table 2:** Execution time and path quality indicators of the optimized trajectory by the proposed method compared to point-to-point operation and RRT* algorithm.

Move	Method	Time [s]	Length [m]	Smoothness [deg]
A 1-2	optimal	6.307	0.4639	$5.16\cdot 10^{-6}$
	manual	6.268	0.4601	0
	RRT*	10.177	0.6823	$4.88\cdot 10^{-6}$
A 2-3	optimal	14.040	1.2263	$3.33\cdot 10^{-6}$
	manual	17.258	1.2158	48.9
	RRT*	23.975	1.291	$2.65\cdot 10^{-6}$
A 3-4	optimal	10.316	0.8649	$3.05\cdot 10^{-6}$
	manual	10.284	0.8617	0
	RRT*	18.356	0.9367	$2.59\cdot 10^{-6}$
A 4-5	optimal	9.299	0.7632	$3.13\cdot 10^{-6}$
	manual	9.299	0.7632	0
	RRT*	15.079	0.9481	$2.57\cdot 10^{-6}$
A 5-6	optimal	2.775	0.1000	$4.15\cdot 10^{-6}$
	manual	2.775	0.1000	0
	RRT*	9.543	0.1304	$3.04\cdot 10^{-7}$
A 6-7	optimal	14.753	1.3072	$5.63\cdot 10^{-6}$
	manual	16.772	1.3439	26.3
	RRT*	31.995	1.773	$2.16\cdot 10^{-6}$
B 1-2	optimal	6.268	0.4601	$5.18\cdot 10^{-6}$
	manual	6.268	0.4601	0
	RRT*	15.815	0.5793	$1.14\cdot 10^{-6}$
B 2-3	optimal	9.299	0.7632	$3.13\cdot 10^{-6}$
	manual	9.299	0.7632	0
	RRT*	24.593	1.0434	$1.43\cdot 10^{-6}$
B 3-4	optimal	14.868	1.3198	$3.03\cdot 10^{-6}$
	manual	18.180	1.3180	65.3
	RRT*	53.097	2.6571	$1.07\cdot 10^{-6}$
B 4-5	optimal	14.215	1.2353	$3.77\cdot 10^{-6}$
	manual	17.305	1.2305	71.1
	RRT*	45.268	2.6218	$4.43\cdot 10^{-6}$
B 5-6	optimal	2.801	0.1027	$4.75\cdot 10^{-6}$
	manual	2.775	0.1000	0
	RRT*	2.775	0.1000	$3.41\cdot 10^{-6}$
B 6-7	optimal	12.225	1.0556	$3.93\cdot 10^{-6}$
	manual	15.595	1.0594	51.1
	RRT*	25.353	1.2630	$1.36\cdot 10^{-6}$

**Table 3:** Comparison of absolute maximum and smoothness values of velocity, acceleration, and jerk during trajectory.

Item	Ind.	Vel. Profile	Vel. [mm/s]	Acc. [mm/s^2^]	Jerk [mm/s^3^]
None	max	Rectangular	114.83	2460.26	110,573.93
		Trapezoidal	100.89	317.13	23,448.88
		S-curve	73.61	217.92	14,414.42
	*S*	Rectangular	0.56	24.05	1779.74
		Trapezoidal	0.14	8.30	746.28
		S-curve	0.09	6.08	558.27
Liquid 0.1 kg	max	Rectangular	112.89	2584.58	113,622.47
		Trapezoidal	101.21	272.49	15,602.73
		S-curve	72.76	204.44	15,168.29
	*S*	Rectangular	0.58	24.83	1821.15
		Trapezoidal	0.13	8.03	734.06
		S-curve	0.09	6.22	566.76
Solid 1 kg	max	Rectangular	115.03	2601.22	119,661.56
		Trapezoidal	100.55	349.51	25,193.59
		S-curve	74.20	201.43	13,912.22
	*S*	Rectangular	0.57	26.26	1974.22
		Trapezoidal	0.15	10.12	920.89
		S-curve	0.10	6.62	605.59
Solid 2 kg	max	Rectangular	114.84	2462.03	127,953.17
		Trapezoidal	101.07	526.19	44,140.82
		S-curve	73.30	231.38	17,695.38
	*S*	Rectangular	0.58	29.46	2357.77
		Trapezoidal	0.18	12.94	1178.53
		S-curve	0.09	6.27	573.34

## Data Availability

Dataset available on request from the authors.

## Supplementary Materials

The following supporting information can be downloaded at: https://www.mdpi.com/article/10.3390/s24165332/s1, Video S1: Robotic Arm profiling and S2: Simulation.
